# Prevalence of Undiagnosed Depression in Patients With Type 2 Diabetes

**DOI:** 10.3389/fendo.2019.00259

**Published:** 2019-05-03

**Authors:** Dina Siddiq Abdulhadi Alajmani, Amna Mohamad Alkaabi, Mariam Waleed Alhosani, Ayesha Abdulaziz Folad, Fawzia Ahmed Abdouli, Frederick Robert Carrick, Mahera Abdulrahman

**Affiliations:** ^1^Dubai Health Authority, Dubai, United Arab Emirates; ^2^Department of Neurology, Carrick Institute, Cape Canaveral, FL, United States; ^3^Department of Neurology, University of Central Florida College of Medicine, Orlando, FL, United States; ^4^Centre for Mental Health Research in Association, University of Cambridge, Cambridge, United Kingdom

**Keywords:** diabetes, depression, mental health, glycemic control, United Arab Emirates

## Abstract

**Introduction:** Type 2 Diabetes Mellitus (T2DM) is the most prevalent type of diabetes among adults and constitutes around 90% of all cases. Substantial evidence demonstrates that depression in the context of diabetes is associated with a wide range of adverse consequences such as reduced adherence to the prescribed treatment regimen, lower quality of life, higher fasting glucose and HbA1c levels, and higher health expenditures.

**Methods:** This study was conducted to assess the depression among T2DM patients attending diabetic clinics, primary healthcare centers (PHC), Dubai Health Authority (DHA). Depressive symptoms were assessed by using both Arabic and English version of the Beck Depression Inventory.

**Results:** Out of 1,050 diabetic patients approached, 559 were within our inclusion criteria and agreed to participate in this study (Response rate of 53%). The mainstream of the participants had T2DM for <10 years (393, 70%), were under oral hypoglycemic treatment only (479, 86%), and had good medication adherence (526, 94%). The overall depression prevalence using a cutoff of 16 was 17%. When we assessed the level of depression amongst participants in association with their sociodemographic and clinical characteristics, there was a significant difference between age groups (*p* < 0.00001); gender (*p* < 0.0001); nationality (*p* < 0.00001); educational level (*p* < 0.00001); and employment status (*p* < 0.0001). The type of clinic in which the T2DM patients were attending (e.g., diabetes mini-clinic vs. General Family Clinic) was also significantly associated with depression (*p* < 0.0001).

**Conclusion:** Our results demonstrate that the intensive service being given in a diabetes mini-clinic compared to routine PHC clinics appears to benefit the psychological aspects of T2DM patients in the UAE population resulting in a lower incidence of depression than commonly seen in a diabetic population. We have identified a need for the establishment of these mini-clinics in each PHC clinics; and the development of campaigns and educational programs, both for health care providers and the public to decrease depression in T2DM patients in this region.

## Introduction

There are differences and similarities between the diabetes-specialty clinics with respect to diabetes management and outcome underscoring the necessity for a protocol-driven treatment approach in ensuring improved diabetes care and outcome (1). We have developed diabetes-specialty clinics in UAE and desired to investigate and differences in outcomes of diabetes treatment in these clinics and in the general medical clinic.

Diabetes mellitus (DM) is a chronic, multifaceted, and progressive disease. Global estimates demonstrate an increase in the prevalence of DM from 422 million patients in 2016 to 642 million by the year 2040 (2). Type 2 DM (T2DM) is the most prevalent type of diabetes among adults and constitutes around 90% of all cases (2–4). Remarkably, 70% of these individuals live in developing countries (5, 6). According to the International Diabetes Federation (IDF), more than 35.4 million of people in the Middle East and North Africa region have diabetes and it is predicted that the number will rise to 72.1 million by 2040 (2).

The UAE is a newly developing country with massive changes in lifestyle and eating habits occurring over the last 47 years. After the discovery of oil in this region in the 1970s, urbanization and growth in prosperity brought major lifestyle changes to the native population. A survey from the United Arab Emirates (UAE) in 2009, found a diabetes prevalence of 23% (7), placing UAE among the countries in the world with the highest diabetes prevalence (2). According to IDF, the prevalence of DM in UAE was 15.6% in the year 2017, and it might reach to 23.4% by 2045; furthermore, there were over 1 million cases of diabetes in UAE in 2017 (2).

Depression is a common comorbidity in individuals with diabetes, compared to those without diabetes (8, 9), affecting approximately 20% of all patients (10, 11). Substantial evidence demonstrates that depression in the context of diabetes is associated with a wide range of adverse consequences such as reduced adherence to a prescribed treatment regimen (11), lower quality of life, higher fasting glucose, and HbA1c levels (12, 13), and higher health expenditures (14). Depressed and anxious individuals are less likely to comply with diabetes self-care recommendations and are more likely to follow a sedentary lifestyle with a probability of poor diabetes control and clinical outcomes. Comorbid depression in diabetic patients can be responsible for premature morbidity, mortality, developing complications, increased pain, and suffering and escalated costs (15).

This study determined the prevalence of depression and its association with socio-demographic and clinical factors in patients attending the Diabetic mini-Clinic located in primary health care centers (PHC) in the Dubai Health Authority. We expect that the findings of this study will be helpful in developing protocols and guidelines to be implemented in PHC to decrease depressive mental illness in the diabetic population in the UAE. Furthermore, the finding of the study can be used as a baseline for other researchers who desire to conduct larger scale studies.

## Methods

### Study Design

This Cross-sectional survey was conducted in Dubai, from December 2017 to November 2018.

### Study Procedure

Participants for the study were recruited from diabetic clinics and primary healthcare centers in the Dubai Health Authority (DHA). Patients with T2DM, between the age of 20 and 65 years and composed of both UAE citizens (nationals) and expatriates, were included in the study. Patients with type I DM, those who had the previous history of psychiatric illness or presently receiving any form of psychiatric treatment, or those who had cognitive impairment or a family history of depression, were excluded. Exclusions were made to avoid confounding due to any effects of ongoing psychiatric treatment. The sample size of our study, cross-sectional design, was calculated using epidemiological information for a population of 8,568 (e.g., total cases of T2DM in PHC for the year 2017 in DHA), with an alpha of 5% maintaining a 95% confidence level with 80% power. We calculated that we needed a minimum required sample size of 368 (16). The participants were assured of the confidentiality of the information provided and the protection of their rights to privacy, mandated by the research ethics guidelines of the human research ethics committees.

### Survey Design (Evaluation Tools)

A structured questionnaire was designed and developed by a multidisciplinary team after thorough review of the literature from relevant studies (11, 17–19). The evaluation tool was then pre-tested among 20 adults to assess ease of understanding and time required for completion. The survey consisted of three functional domains: socio-demographic characteristics, DM data, and depression analysis. The demographic data included age, gender, nationality, marital status, educational level, occupation, and employment status. Diabetic data included the duration of DM, type of treatment, practice of exercise, smoking, glycemic control, presence of any comorbidities, and DM complications. Depressive symptoms were assessed using both the Arabic and English version of the Beck Depression Inventory (BDI) (20, 21). The Beck Depression Inventory (BDI) contains 21 multiple-choice questions, and each option has a range of 0–3 scores; higher scores indicate the severity of depression. This questionnaire has a maximum score of 63 points in which 0–10 represents a normal, 11–16 indicates mild mood disturbance, 17–20 borderline clinical depression, 21–30 indicates moderate depression, and 31–63 indicates severe depression. The BDI demonstrates high internal consistency with a mean coefficient alpha of 0.86 reported for psychiatric groups and 0.81 for non-psychiatric groups (22). Questionnaires were administered during face-to-face interviews conducted in Arabic or English by physician researchers.

### Data Analysis and Statistics

Statistical analysis of the data was performed using STATA 14 (StataCorp College Station, TX), maintaining an alpha (α) 0.05 with 80% power to decrease the possibility of making Type 1 and 2 errors. Descriptive statistics were computed for the socio-demographic variables. The overall responses to each item of the survey were recorded as a percentage of the total. The percentage differences in the total responses were determined using the Chi-square test and statistical significance recorded for non-parametric data. For all tests, linear and logistic regression models were fitted to search for any statistically significant predictors of diabetes and depression in the sample. A linear mixed model exploring the total diabetic score with each individual clinic was fit and estimated with a restricted maximum likelihood (REML) approach. Correlations between variables were calculated, and the discrimination of fitted logistic models was analyzed.

### Ethics Statement

The study was approved by the institutional review boards of Dubai Health Authority, Dubai (Ethics approval # DSREC/RRP/2017/22). Participants were not compensated, and all participants gave informed consent before participation. Aggregate reporting of data was assured to enhance confidentiality and accurate reporting by the respondents. The return of the completed survey also guaranteed the anonymity of participation constructs to an administrator; independent and blinded to the study hypothesis. A code linking respondents to their surveys was kept isolated from the investigators.

## Results

Out of 1,050 diabetic patients approached, 559 were within our inclusion criteria and agreed to participate in this study (Response rate of 53%). The interview was conducted both in the Diabetes Mini-Clinic and the General Family Clinic, and participants were recruited equally in both (45% vs. 55%, respectively). The majority of our T2DM respondents were female (318, 57%), aging >50 years (366, 65%), UAE national (412, 74%), married (504, 90%), having high school or less certificate (453, 81%), and unemployed (293, 52%), Table 1.

**Table 1:** Descriptive demographic characteristics of type 2 diabetic patients (*n* = 559).

**Variable**	***n* (%)**
**Gender**	
Female	318 (57)
Male	241 (43)
**Age (years)**	
21–30	4 (1)
31–40	52 (9)
41–50	137 (25)
51–60	248 (44)
>60	118 (21)
**Nationality**	
UAE	412 (74)
Non-UAE	147 (26)
**Marital status**	
Single	31 (6)
Married	504 (90)
Divorced/widowed	24 (4)
**Education level**	
Less than high school	245 (44)
High school	208 (37)
Diploma or Bachelor's degree	93 (17)
Post Grad or Higher	13 (2)
**Employment status**	
Employed	266 (48)
Unemployed	293 (52)

The largest number of patients (384 subjects) representing 68.69% of subjects were seen in one of the eleven (11) primary healthcare centers (PHC) involved in this study, Table 2. We did not find any difference in the type of patients seen between each of the 11 PHCs in our study. A linear mixed model exploring the total diabetic score with each individual clinic was fit and estimated with a restricted maximum likelihood (REML) approach. We estimated the fixed effects as *b*_0 = 8.27 and *b*_1 = −0.21. The estimated variance components revealed a Sigma∧u was estimated as 0.00 with a standard error of 0.00. We obtained a Wald test (0.45) comparing the coefficient's estimated value with the estimated standard error for the coefficient where the coefficient's estimate was expected to be normally distributed (*z*-test = −0.67, *P* = 0.50). A likelihood-ratio test comparing the model to ordinary linear regression is highly significant for these data (LR test vs. linear model: chi2(2) = 21.21; *P* > chi2 = 0.00) Table 3.

**Table 2:** Patient distribution per primary health care clinic.

**PHC**	**Frequency**	**Percent**	**Cumulative**
1	384	68.69	68.69
2	25	4.47	73.17
3	6	1.07	74.24
4	3	0.54	74.78
5	2	0.36	75.13
6	46	8.23	83.36
7	30	5.37	88.73
8	2	0.36	89.09
9	49	8.77	97.85
10	8	1.43	99.28
11	4	0.72	100.00
Total	559	100.00	

**Table 3:** Mixed-effects ML regression.

**Diabetes total score**	**Coef**.	**Std. err**.	***Z***	***P***	**95% conf. interval**
PHC	−0.2072795	0.3093964	−0.67	0.503	−0.8136852, 0.3991262
cons	8.271454	2.011414	4.11	0.000	
**Number of observations** **=** **559, Number of groups** **=** **11**					
**Random-effects parameters**	**Estimate**	**Std. err**.	**95% conf. interval**		
**Clinic:Independent**					
Sd (Clinic)	0.0005027	0.0036905	2.84e-10, 890.8998		
Sd (cons)	2.554172	0.8453436	1.335155, 4.886172		
Sd (Residual)	6.896664	0.2079046	6.500982, 7.316428		

The mainstream of the participants had T2DM for <10 years (393, 70%), were under oral hypoglycemic treatment only (479, 86%), had good medication adherence (526, 94%), were not smokers (494, 88%), but were not practicing exercise (284, 51%). However, although the majority of the participants assumed that their glycemic control was poor (270, 48%), their HbA1c was well controlled (294, 53%), and they were attending the diabetic clinic at least once every 3 months (416, 75%). The vast majority of participants did not have any DM complications (482, 86%); Table 4 shows types of comorbidities and diabetes complications among contributors.

**Table 4:** Clinical characteristics of type 2 diabetic (T2DM) patients participated in this study, *n* = 559.

**Variable**	***n* (%)**
**Where the interview conducted**	
Mini Diabetes Clinic	249 (45)
General Family Clinic	310 (55)
**How long you are diagnosed with T2DM**	
1–10 years	393 (70)
11–20 years	126 (23)
>20 years	40 (7)
**Type of treatment**	
None or diet only	6 (1)
Oral hypoglycemic only	479 (86)
Insulin only	8 (1)
Insulin and oral hypoglycemic	66 (12)
**Medication adherence (e.g., taking your medications)**	
**regularly**	
Yes	526 (94)
No	33 (6)
**Do you exercise**	
Yes	275 (49)
No	284 (51)
**Do you smoke**	
Yes	65 (12)
No	494 (88)
**How is you glycemic control**	
Poorly controlled (HbA1c ≥7)	270 (48)
Well controlled (HbA1c <7)	247 (44)
I don't know	42 (8)
**Latest HbA1c**	
Poorly controlled (HbA1c ≥7)	265 (47)
Well controlled (HbA1c <7)	294 (53)
**How often do you attend your diabetes clinic**	
Every 3 months	416 (75)
Every 6 months	69 (12)
Once a year	30 (5)
First time	44 (8)
**Do you have any other chronic medical illness**	
No	
Yes^*^	482 (86)
Hypertension	38 (7)
Dyslipidemia	151 (27)
Ischemic heart disease	5 (1)
Dyslipidemia and hypertension	208 (38)
Dyslipidemia and ischemic heart disease	11 (2)
**Complication of diabetes**	
None	482 (86)
Peripheral neuropathy	30 (5)
Ischemic heart disease	11 (2)
Eye involvement	24 (4)
Kidney involvement	14 (2)
Delayed wound healing	7 (1)
Amputation	1 (0.1)
Others (obesity, gastroparesis, erectile dysfunction)	4 (0.5)
**Levels of depression**	
Normal	330 (59)
Mild mood disturbance	135 (24)
Borderline clinical depression	46 (8)
Moderate depression	45 (8)
Severe depression	3 (0.5)
Extreme depression	0

* *Respondents were given the choice to mention more than one complication*.

**Figure 1:**
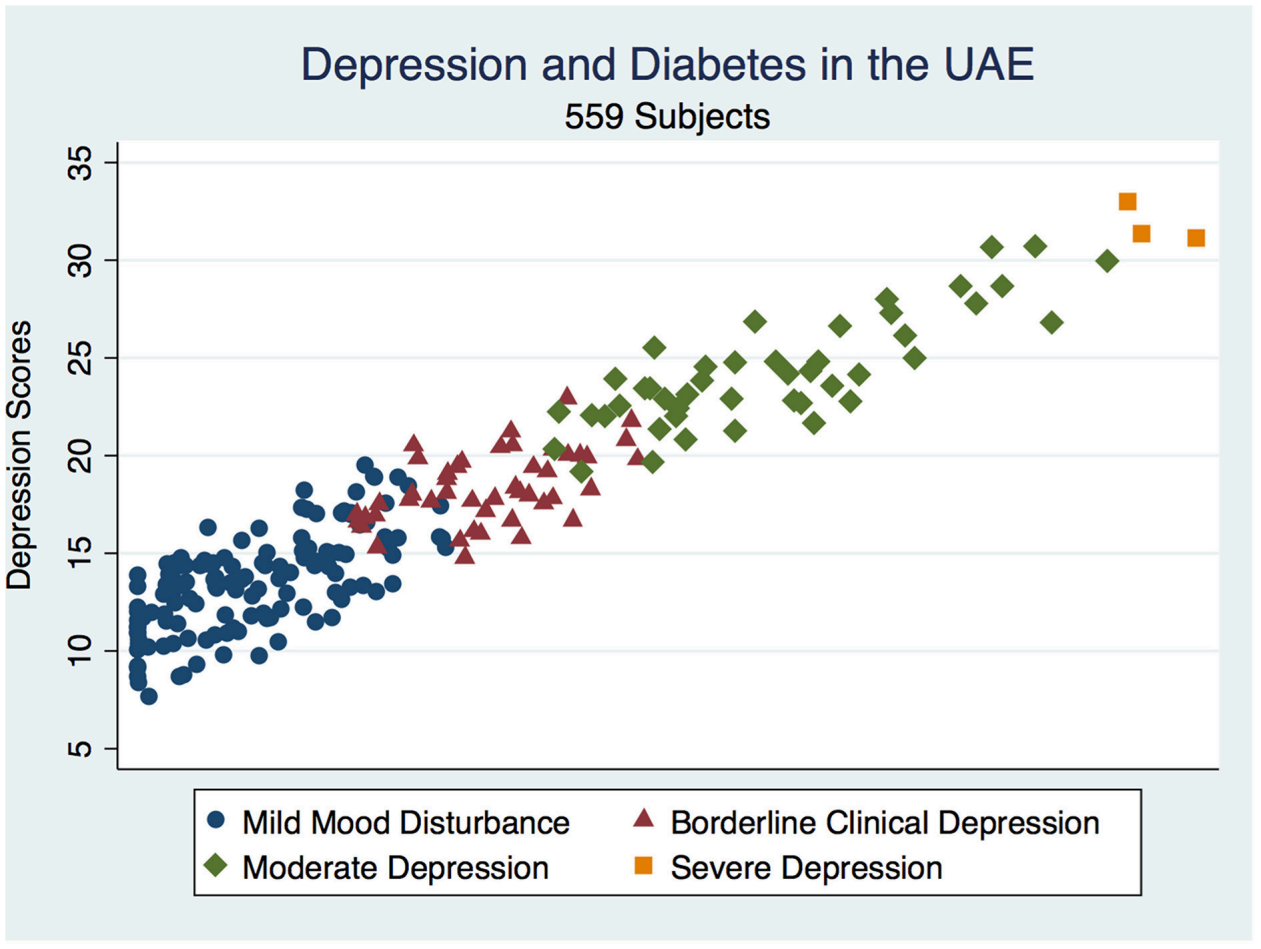
Depicts a scatterplot of the distribution of depressive illness in a diabetic population in the UAE.

**Figure 2:**
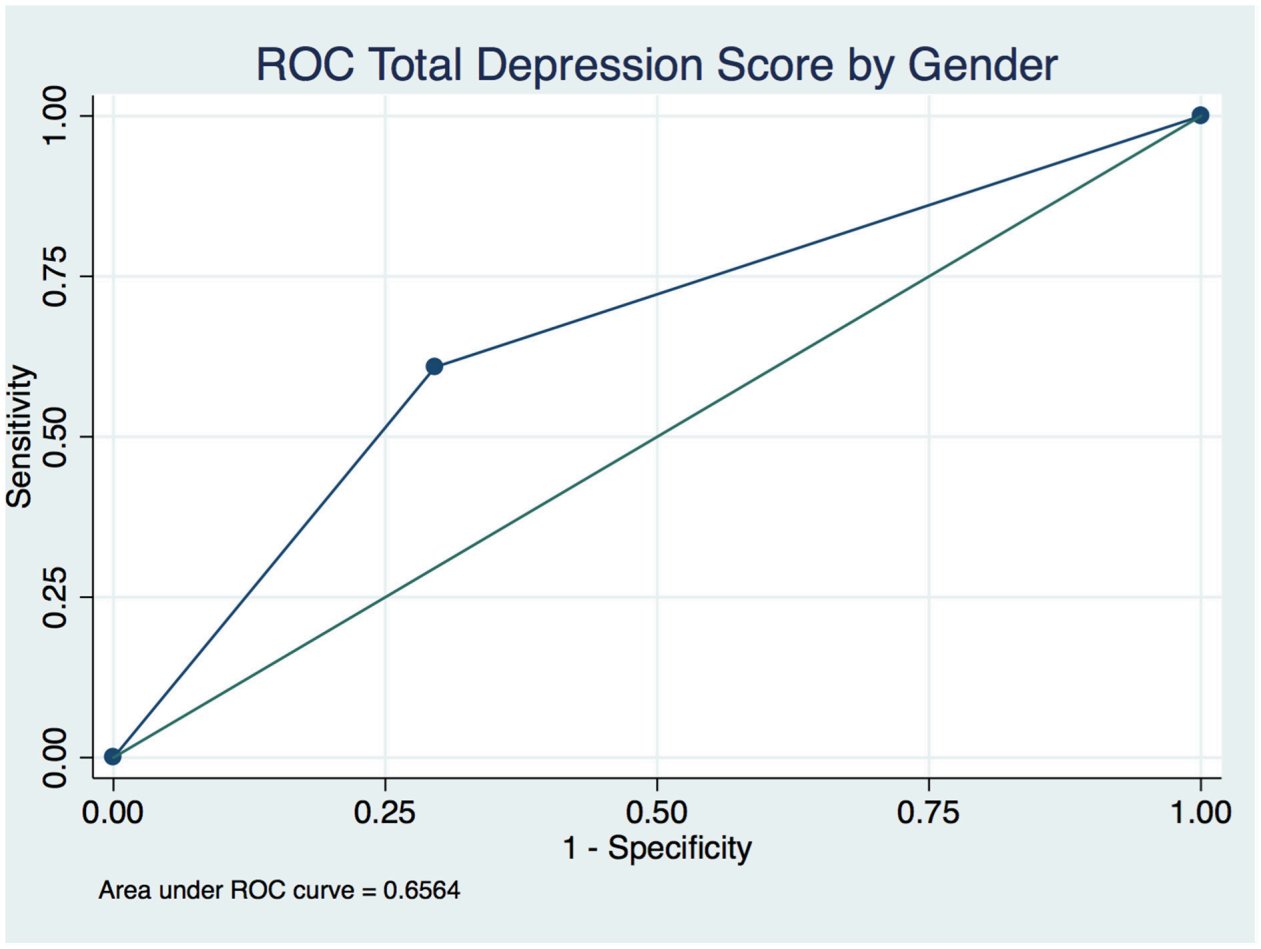
Receiver operating characteristic (ROC) curve of total depression in type 2 diabetic patients by gender.

**Figure 3:**
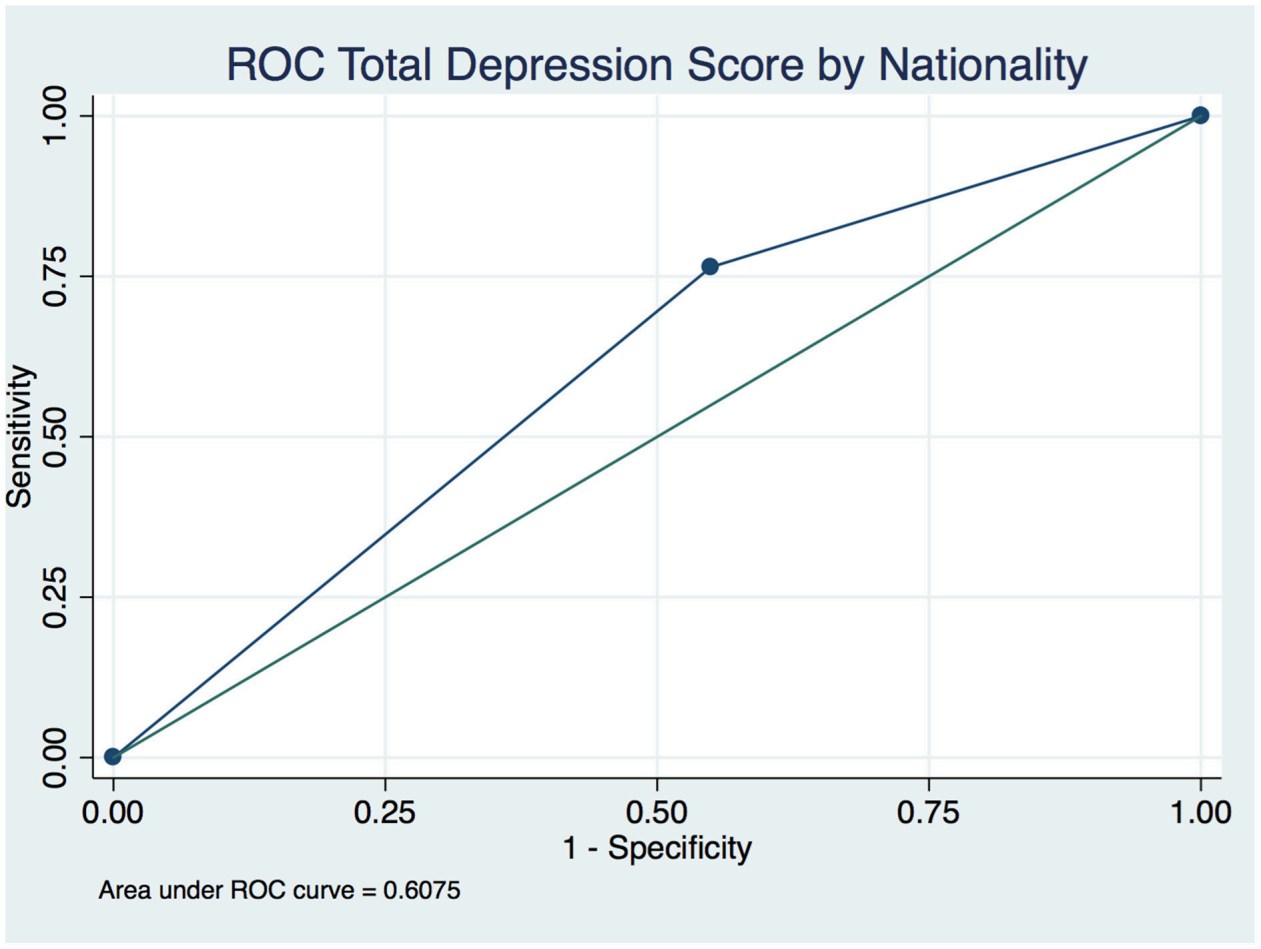
Receiver operating characteristic (ROC) curve of total depression in type 2 diabetic patients by nationality.

**Figure 4:**
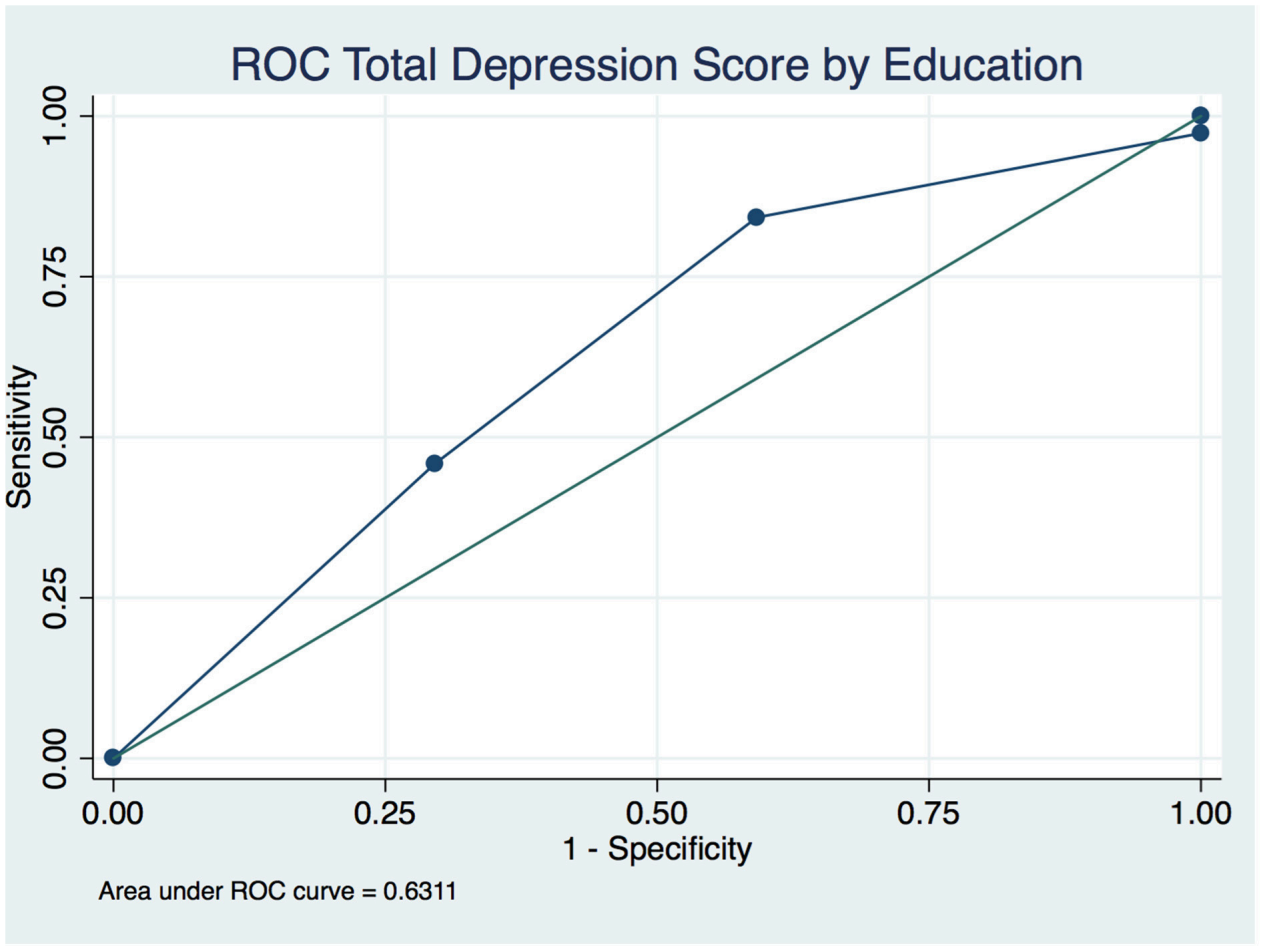
Receiver operating characteristic (ROC) curve of total depression in type 2 diabetic patients by level of education.

The overall depression prevalence using a cutoff of 16 (23) was 17% (Figure 1). When we assessed the level of depression amongst participants in association with their sociodemographic and clinical characteristics, there was a significant difference between different aged groups (*p* < 0.00001); gender (*p* < 0.0001); nationality (*p* < 0.00001); educational level (*p* < 0.00001); and employment status (*p* < 0.0001) (Table 5). The discrimination of fitted logistic models, via receiver operating characteristic (ROC) curves of the total depression score and the gender, nationality and education of all subjects revealed good discrimination values. (Figures 2–4). Although none of the clinical factors were significantly associated with a higher risk of depression, the type of clinic in which the T2DM patients were attending (e.g., diabetes mini-clinic vs. General Family Clinic) was significantly associated with depression (*p* < 0.0001) (Table 5).

**Table 5:** The relationship between sociodemographic characteristics and depression status among T2DM Patients in UAE (*n* = 559).

**Variable**	**Normal (score** **≤10)**		**Mild mood disturbance** **(score 11–16)**		**Borderline clinical depression** **(score 17–20)**		**Moderate depression** **(score 21–30)**		**Severe depression** **(score 31–40)**		***P-Value***
	***n* %**	**Total**	***n* %**	**Total**	***n* %**	**Total**	***n* %**	**Total**	***n* %**	**Total**	
**AGE**											
21–30	1	330	1	135	1	46	1	45	0	3	0.00001
31–40	31		12		5		3		1		
41–50	84		32		10		11		0		
51–60	147		61		21		19		0		
>60	67		29		9		11		2		
**GENDER**											
Male	163 (49)	330	43 (32)	135	20 (43)	46	15 (33)	45	0		0.0001
Female	167 (51)		92 (68)		26 (57)		30 (67)		3 (100)	3	
**NATIONALITY**											
UAE	226 (68)	330	106	135	37 (%)	46	40 (%)	45	3 (100)	3	0.00001
Non UAE	104 (32)		29		9 (%)		5 (%)		0		
**EDUCATION LEVEL**											
Less than high school	136 (41)	330	59 (44)	135	25 (54)	46	23 (51)	45	2 (67)	3	0.00001
High school	115 (35)		56 (42)		16 (35)		20 (45)		1 (33)		
Diploma/bachelor degree	74 (23)		14 (10)		4 (9)		1 (2)		0		
Postgraduate/higher degrees	5 (1)		6 (4)		1 (2)		1 (2)		0		
**EMPLOYMENT STATUS**											
Employed	172(52)	330	54 (40)	135	23 (50)	46	16 (35)	45	1 (33)	3	0.0001
Unemployed	158 (48)		81 (60)		23 (50)		29 (65)		2 (67)		
**CLINIC**											
Diabetes mini-clinic	165	330	60	135	13	46	9	45	2	3	0.0001
PHC^*^ clinic	165		75		33		36		1		

* p < 0.05, significance determined using Montecarlo 2 tailed significance at 95% CI. Only significant results are presented. *Primary health care clinic.

## Discussion

Depressive symptoms that are not severe enough to warrant a diagnosis of clinical depression are highly prevalent in the diabetic population and are associated with both poor well-being and poor diabetes self-management (24). A cutoff of 16 or higher on the Beck Depression Inventory was suggested to be accurate in diabetes (23), representing an overall depression prevalence of 32.8% in diabetes (25). The overall prevalence of depression (borderline, moderate, and severe depression) in our studied population was much less at 17% (94/559). We did not find any difference in the type of patients seen between each of the 11 PHCs in our study. We compared the prevalence of depression in our sample to that of patients with T2DM throughout the world: 43.5% in Pakistan (5), 40% in Palestine (26), 7.8% in Nigeria (27), 13–61% in Ethiopia (28), and 37% in Turkey (29). A meta-analysis of 42 studies found that approximately 20–40% of individuals with T2DM have comorbid depression, a prevalence at least double that found in the general population (30). Hence, our results of 17% seem to show a lower rate of depression compared to worldwide reports.

Our study shows a higher prevalence of depression in T2DM females than males (14.5 vs. 18.5%, *p* < 0.0001); this is in agreement with other studies reported worldwide (31–33). We found a significant association between depression prevalence in T2DM patients and their education level and employment status (those not employed and with less education had higher rates of depression compared to their counterparts (*p* < 0.0001 and *p* < 0.00001, respectively). Other reports also revealed that a higher level of educational standard attained has a protective effect against both anxiety and depression (34, 35). We realized that patients with a low level of education may leave some questions unanswered during written administration of this questionnaire. We intentionally avoided this problem by administering it during direct face-to-face interviews. We found that the clinic in which the patient was getting treatment was a modifiable risk factor that contributed to depression. The mini-diabetes clinic is a specialist clinic located in some of the primary health care centers, initiated in Dubai health authority since 2004 and staffed by family physicians providing comprehensive medical care to diabetic patients through a multidisciplinary team approach specific to diabetes. In addition to the family physician, the team at each mini-diabetes clinic consists at least of a registered nurse, a health educator, a nutritionist, and a pharmacist. The criteria to refer patients with type 2 diabetes mellitus from family medicine clinics to mini-diabetes clinics are as follows: HbA1c ≥7% on 2 consecutive visits apart for 3 months despite proper management; elderly patients (65 years and above) with HbA1c ≥7.5% on 2 consecutive visits apart for 3 months despite proper management. All patients with T2DM are required to attend the clinic once per year for comprehensive diabetes care. The uniqueness of the diabetes mini-clinic includes the time allocated to the patient: 40 min vs. 15 min in ordinary PHC clinics. This allows more time for comprehensive examination and encourages the patients to participate in the decision-making process while enhancing the patient's concordance with medical care regimens. Another difference is that, at every visit, the patient undergoes an extensive complete review of his/her diabetic wellbeing. The services provided include weight and body mass index assessment, colored fundus photography, foot care, cardiovascular risk assessment with Electrocardiography if appropriate, review of smoking habits and renal functions, immunization status, as well as medication review and diet education. Retinal camera screening, which started in PHC in April 2015 is obtained at least annually for all diabetic patients registered in PHC facilities. Our results suggest that the intensive service being given to the T2DM patients in diabetes mini-clinics in PHC is not only comprehensive in the clinical service and diagnosis but it also appears to affect the long-term improvement of the psychological outcome of the patients. Further investigation is warranted to clarify possible mechanisms of improvement.

## Conclusions

This study is the first to examine depression in patients with T2DM in the United Arab Emirates. Our results demonstrate that the intensive service being given in diabetes mini-clinics compared to routine PHC clinics benefits the psychological aspects of T2DM patients in the UAE population and is associated with a lower incidence of depressive mental illness. There is a need for the establishment of these mini-clinics in each PHC clinic and the development of more campaigns and educational programs, both for health care providers and public on depression in T2DM patients. We recommend that healthcare administration and public health policymakers in the UAE should utilize more educational tools, in order to increase the awareness of the community on the risk of depression in diabetes patients. Finally, we suggest that new policies need to be established that focus more on increasing community awareness of diabetes and depression preventive measures in UAE.

## Limitations

This study has several limitations. It focused on patients in governmental sectors in Dubai, UAE so it may not be generalized to all populations. Therefore, the results of this study should be interpreted with caution. This study used a cross-sectional design that speculates on the causal relationship between the variables. It used convenience sampling so that the results might be unrepresentative of the general population. However, despite these limitations, the results of this study provide a basis for further planning and future in-depth research needs to assist in the development of educational materials and planning training-based interventions for further boosting psychological care and clinical management of patients with T2DM in UAE.

## Ethics Statement

The study was approved by the institutional review boards of Dubai Health Authority, Dubai (Ethics approval # DSREC/RRP/2017/22). Participants were not compensated, and all participants gave informed consent before participation. Aggregate reporting of data was assured to enhance confidentiality and accurate reporting by the respondents. The return of the completed survey also guaranteed the anonymity of participation constructs to an administrator; independent and blinded to the study hypothesis. A code linking respondents to their surveys was kept isolated from the investigators.

## Author Contributions

All authors listed have made a substantial, direct and intellectual contribution to the work, and approved it for publication.

### Conflict of Interest Statement

The authors declare that the research was conducted in the absence of any commercial or financial relationships that could be construed as a potential conflict of interest.
